# Causal network analysis-based assessment of gray matter alteration in post-radiotherapy nasopharyngeal carcinoma patients using 3D T1-weighted MRI

**DOI:** 10.3389/fnins.2026.1709659

**Published:** 2026-04-13

**Authors:** Chunhong Qin, Huan Lin, Yujie Liu, Hongzhuo Wang, Jie An, Fuhong Duan, Donglin Wu, Qianli Wang, Shijun Qiu, Xi Leng

**Affiliations:** 1 Department of Radiology, The First Affiliated Hospital of Guangzhou University of Chinese Medicine, Guangzhou, China; 2 Department of Radiology, Guangdong Provincial People's Hospital, Guangdong Academy of Medical Sciences, Guangzhou, China

**Keywords:** 3D T1-weighted magnetic resonance imaging, causal structural covariance network, cognitive function, nasopharyngeal carcinoma, radiation induced brain injury

## Abstract

**Objectives:**

To explore the temporal and causal relationships underlying brain structural changes in post-radiotherapy (RT) nasopharyngeal carcinoma (NPC) patients.

**Methods:**

A total of 38 post-radiotherapy NPC patients (33 males, 5 females; median age: 50.0 years, range: 27–63 years; median time post-RT: 17.2 months, range: 0.5–108 months) and 23 healthy controls (16 males, 7 females; median age: 37 years, range: 24–61 years) underwent T1-weighted magnetic resonance (MR) images, and their images were evaluated. The causal structural covariance network (CaSCN) analysis approach was applied to assess the causal relationships underlying radiation-induced brain structural alterations in these patients. Granger causality (GC) analysis was employed to morphometric data derived from T1-weighted MR images, which were ordered by the time elapsed post-RT.

**Results:**

The source-like directed associations were observed in the bilateral parahippocampal gyrus (PHG), the right gyrus rectus (REC.R), and the right caudate nucleus (CAU.R). The directed network analysis revealed that the parahippocampal gyrus (PHG), REC.R and CAU.R exhibited typical source-like characteristics, and their structural changes exerted a key regulatory effect on GM volume alterations across multiple brain regions. While the left precuneus (PCUN.L), left temporal pole: middle temporal gyrus (TPOmid.L) and the left inferior temporal gyrus (ITG.L) were typical sink-like brain region that mainly received regulatory effects from source-like brain regions, acting as major target regions of structural damage.

**Conclusion:**

Over time, post-radiotherapy NPC patients exhibited progressive changes in GM volume, where the PHG.L, PHG.R, REC.R and CAU.R were core source-like brain regions. The PCUN.L, TPOmid.L, and ITG.L show distinct sink-like features, which mainly receive regulatory effects from source-like brain regions.

## Introduction

Nasopharyngeal carcinoma (NPC) represents a highly prevalent malignancy of the head and neck region in southern China and Southeast Asia (Li et al., 2024). Although radical radiotherapy serves as the primary treatment, it can cause severe central nervous system complications referred to as radiation-induced brain injury (RBI), which substantially impairs patients' quality of life (Huang et al., 2025). RBI not only damages white matter (WM) structures but also leads to gray matter (GM) impairment. Post-radiotherapy GM volume reduction has been conclusively demonstrated (Lv et al., 2014), yet the affected regions extend beyond the temporal lobes closest to the radiation field, manifesting as a widespread and dynamic degenerative process that involves the frontal lobes, parietal lobes, cerebellum, and other structures (Leng et al., 2017). The underlying pathophysiological mechanisms remain elusive. The prevailing hypothesis posits that RBI exhibits progressive deterioration in both brain morphology and functional connectivity over time following radiotherapy (RT). Therefore, investigating the interregional relationships of progressive brain damage is of paramount importance for elucidating the pathogenic mechanisms underlying RBI.

The advent of structural covariance network (SCN) analysis has enabled the quantitative assessment of correlated morphometric changes among brain regions (Evans, 2013), and this method is now widely used to investigate network properties and structural covariance patterns in various diseases (Zugman et al., 2015). This approach, however, is inherently limited: it relies on correlation analysis (specifically zero-time-lag correlation) to infer synchrony, and it is unable to establish causal relationships across brain regions or identify the temporal precedence of damage between one region and another (Jiang et al., 2018).

As a widely-used alternative, Granger causality (GC) analysis delineates the directionality of information flow by detecting sequential patterns in neural activity across regions and can also predict regional brain activity. It has played a pivotal role in analyzing functional time-series data across various neuropsychiatric disorders, including schizophrenia, social anxiety disorder, and epilepsy (Chen et al., 2017; Luo et al., 2016). By organizing morphometric data based on temporal variables—such as disease progression and post-treatment duration—and subsequently applying Granger causality (GC) analysis, researchers can investigate causal relationships underlying structural changes across brain regions. Referred to as causal structural covariance network (CaSCN) analysis, this integrated approach has been effectively utilized in research on a range of central nervous system diseases (Zhang et al., 2017). However, few studies have investigated the causal relationships underlying network alterations in RBI. To address this research gap, we hypothesize that CaSCN can effectively characterize progressive changes in structural networks among RBI patients. This study systematically delineates the evolutionary trajectory of structural networks throughout the progression of RBI and employs CaSCN analysis to rigorously assess causal effects underlying structural alterations in RBI patients. To our knowledge, it is the first investigation to dissect causal relationships in RBI-related structural damage.

## Materials and methods

### Participants

This study was approved by the Ethics Committee of the First Affiliated Hospital of Guangzhou University of Chinese Medicine. All procedures were strictly conducted in accordance with the principles of the Declaration of Helsinki and complied with the approved ethical standards. Each participant signed a written informed consent form prior to participating in the study. A total of 38 patients with pathologically confirmed NPC and 23 healthy controls (HCs) were enrolled from the First Affiliated Hospital of Guangzhou University of Chinese Medicine in the study. All patients received initial intensity-modulated radiotherapy (IMRT) with a total dose of 66–74 Gy, a single fraction dose of 1.8–2.0 Gy, administered in 30–35 fractions. All patients received standard platinum-based chemotherapy in combination with IMRT according to institutional treatment protocols. Before magnetic resonance imaging (MRI) examination, all patients were confirmed to have no evidence of intracranial tumors or intracranial invasion. Individuals with comorbidities such as hypertension, diabetes mellitus, heart disease, white matter degeneration, or vascular lesions were excluded. Healthy volunteers served as controls and were screened using the same criteria as the patients with NPC post-RT. Statistical analyses were performed using SPSS 20.0 software (SPSS Inc., Chicago, IL, USA). Differences in gender were compared using the chi-square test. For continuous variables (age and educational level), normality was first assessed *via* the Shapiro–Wilk test. For variables that follow a normal distribution, the two-sample t-test was used for intergroup comparisons, whereas for variables that do not conform to a normal distribution, the nonparametric Kruskal–Wallis test was employed. A *P*-value < 0.05 was considered statistically significant, with the false discovery rate (FDR) applied to correct for multiple comparisons.

### Cognitive assessment

All participants completed a standardized neuropsychological battery that included: (1) the Montreal Cognitive Assessment–Basic (MoCA-B); (2) the Auditory Verbal Learning Test (AVLT; Zhao et al., 2015), assessing immediate recall, short-term delayed recall, long-term delayed recall, and recognition; (3) the Trail Making Test (TMT, Parts A and B; Muir et al., 2015); (4) the Digit Symbol Substitution Test (DSST; Chen et al., 2020); and (5) the Digit Span Test (DST, both forward and backward; Diamond, 2013). Administration required a minimum of 35 min. Since significant intergroup differences in age and education level were identified, these variables were incorporated as covariates in subsequent regression analyses examining cognitive performance The statistical significance threshold was set at *p* < 0.05, with the FDR applied to correct for multiple comparisons.

### Image acquisition

All MRI data were acquired using a 3.0 T clinical scanner (SIGNA EXCITE; GE Healthcare, Chicago, IL, USA). First, conventional clinical sequences—including T1-weighted, T2-weighted, and T2-FLAIR images—were obtained to confirm the absence of detectable brain lesions. Subsequently, high-resolution three-dimensional (3D) T1-weighted images were acquired with the following parameters: repetition time (TR) = 5.5 ms, echo time (TE) = 1.5 ms, flip angle = 12°, acquisition matrix = 256 × 256, field of view (FOV) = 256 × 256 mm^2^, number of sagittal slices = 166, slice gap = 0 mm, and voxel size = 1 × 1 × 1 mm3. The 3D T1-weighted sequence took 5 min and 50 s, with the scan coverage encompassing the entire brain. During the scan, the participant was fitted with noise-reducing earplugs and instructed to remain still to minimize the impact of motion artifacts on image quality.

### Data preprocessing

All high-spatial-resolution 3D T1-weighted images were processed using a standardized voxel-based morphometry (VBM) pipeline implemented in the Computational Anatomy Toolbox (CAT12; https://neuro-jena.github.io/cat/) within Statistical Parametric Mapping (SPM12; https://www.fil.ion.ucl.ac.uk/spm/software/spm12/), running in MATLAB. Prior to preprocessing, all T1-weighted images were visually inspected for artifacts (e.g., motion, ghosting, incomplete brain coverage, or severe intensity inhomogeneity). Images were manually reoriented with the anterior commissure (AC) set as the origin of the coordinate system. VBM preprocessing consisted of the following steps: (1) Bias-field correction and tissue classification: images were corrected for intensity non-uniformity and segmented into gray matter (GM), white matter (WM), and cerebrospinal fluid (CSF) using CAT12's tissue probability maps with adaptive maximum a posteriori (AMAP) estimation and partial volume estimation; (2) Spatial normalization: individual GM maps were nonlinearly registered to the Montreal Neurological Institute (MNI) space using DARTEL-based high-dimensional normalization; (3) Modulation: modulated GM maps were generated to preserve regional GM volume by correcting for volume changes introduced during normalization (i.e., Jacobian modulation); (4) Smoothing: the modulated GM maps were smoothed with an 8-mm full-width at half-maximum (FWHM) Gaussian kernel. In addition, CAT12′s built-in image quality assessment (IQR; “weighted overall image quality rating”, 0–100) was used as an objective QC criterion. Only images with an overall quality rating >80 were retained for further analysis; images not meeting this threshold were excluded. Finally, the smoothed, modulated GM images were used for group-level voxel-wise GM volume comparisons. Total intracranial volume (TIV) estimated by CAT12 was extracted for each participant and was included as a covariate in subsequent statistical analyses when appropriate.

### Voxel-based morphometry analysis

To characterize changes in whole-brain GM volume in NPC patients post-RT, this study used the two-sample *t*-test integrated in SPM12 for intergroup comparisons, with covariates including gender, age, educational level, total intracranial volume (TIV), and time post-RT controlled for. After applying family-wise error (FWE) correction, statistical significance was set with a voxel-level threshold of *P* < 0.001 and a cluster-level threshold of *P* < 0.05. Furthermore, Spearman correlation analysis was performed to examine the association between GM volume changes and cognitive function in both NPC patients HCs, with the statistical significance level set at *P* < 0.05 and corrected for multiple comparisons using the FDR.

### Voxel-wise causal structural covariance networks analysis

In line with the VBM-based CaSCN analytical pipeline used in prior studies (Jiang et al., 2018; Zhang et al., 2017), a seed-based CaSCN was constructed, with the seed region obtained from the VBM analysis conducted earlier. GM volume data from all post-radiotherapy NPC patients were sorted in ascending order according to post-RT time, which was treated as “pseudo-time series” data to capture the progressive property of RBI. Voxel-wise signed-path coefficient Granger causality (GC) analysis with a first-order model (order = 1) was subsequently performed across the whole brain using the REST MRI toolkit (http://www.restfmri.net/forum/). Gender, age, educational level, TIV, and the time interval between the two pseudo-time points (i.e., irregular time intervals between the participants) were regressed as covariates in the CaSCN analysis. To assess statistical significance, the GC map was converted into a z-score mapstatistical significance was defined at a voxel-level threshold of *P* < 0.001 and a cluster-level threshold of *P* < 0.05, corrected for FDR. Voxels surviving FDR correction (*q* < 0.05) were identified using the joint criterion *Z* > 3.75 and GC > 0.39, where *Z* > 3.75 represents the *Z*-statistic threshold corresponding to *q*(FDR) < 0.05, and GC > 0.39 was used as an additional magnitude threshold. In the Granger-causality-based directed network, the sign of the GC coefficient (and its corresponding *Z* value) reflects the direction of the regression relationship under the pseudo-time ordering. A positive *Z* value indicates that higher GMV in the source region predicts higher GMV in the target region at the next pseudo-time point (i.e., same-direction association), whereas a negative Z value indicates an inverse association (i.e., higher GMV in the source predicts lower GMV in the target). Importantly, the Z sign should be interpreted as a statistical directionality in the model rather than direct neurophysiological excitation/inhibition. This study performed both seed-to-map and map-to-seed GC analyses. The map refers to the Anatomical Automatic Labeling (AAL) template, which includes cerebellar parcellations.

### ROI-wise causal structural covariance networks analysis

For a deeper investigation of causal effects among regions of interest (ROIs) derived from voxel-wise CaSCN analysis, ROI-to-ROI GC analysis was performed following the method established by Zhang et al. (2017). GC analysis with signed path coefficients was adopted to construct an ROI-wise causal network that characterizes interregional causal relationships. For consistency with the voxel-wise CaSCN analysis, the same significance threshold (GC > 0.39) was applied. The binarized “out-degree” and “in-degree” values were computed separately for each ROI in the causal network. Specifically, the in-degree of an ROI is defined as the total number of paths projecting into the ROI, while the out-degree reflects the total number of paths projecting from the ROI to other nodes. Furthermore, the out-in degree (out-degree minus in-degree) was calculated to identify the causal targets or causal source levels of each ROI. Given that the ROI-wise bivariate GC analysis was highly sensitive to indirect pathways and common drivers, we further conducted a multivariate GC analysis to investigate the robustness of the findings.

## Results

### Clinical characteristics and cognitive assessment

While no significant difference was observed in gender distribution between the two groups, significant differences existed in terms of age and educational level. Cognitive performance differed significantly across all neuropsychological measures. Detailed clinical characteristics and cognitive scores were presented in Table 1. In the subsample matched for age, sex, and education level, statistically significant intergroup differences were found for the MoCA-B, AVLT (immediate), AVLT (20 min), TMT-B, and DSST. In contrast, no significant intergroup differences were observed for the AVLT (5 min), AVLT (recognition), TMT-A, and the DST. Detailed clinical characteristics and cognitive scores were presented in Supplementary Table S1. The above results suggested that the intergroup differences observed in some of the cognitive scores may have arisen from disparities in age and education level.

**Table 1:** Clinical characteristics and cognitive assessment of post-radiotherapy NPC patients and HCs.

Clinical information	NPC patients (*n* = 38)	HCs (*n* = 23)	Statistics	*P*-value	FDR-corrected
Clinical characteristics					
Age (years)	50.00 (41.25, 53.75)	37.00 (28.50, 52.00)	*U* = 582.50	0.031	0.046^*^
Sex (M/F)	33/5	16/7	χ^2^ = 1.72	0.189	0.189
Education level (years)	12.00 (9.00, 15.00)	15.00 (12.00, 16.00)	*U* = 283.00	0.019	0.046^*^
Cognitive scores					
MoCA-B	26.00 (25.00, 27.00)	28.00 (27.00, 29.00)	*U* = 164.00	< 0.001	< 0.001^*^
AVLT (immediate)	20.083 ± 0.58	25.354 ± 0.86	*t* = −4.86	< 0.001	< 0.001^*^
AVLT (5 min)	8.00 (7.00, 9.75)	10.00 (8.50, 11.00)	*U* = 240.00	0.003	0.006^*^
AVLT (20 min)	8.00 (7.00, 9.00)	10.00 (8.50, 11.00)	*U* = 193.00	< 0.001	< 0.001^*^
AVLT (recognition)	10.50 (9.25, 12.00)	12.00 (11.50, 12.00)	*U* = 296.50	0.024	0.025^*^
TMT-A	43.95 (32.07, 57.90)	29.96 (25.20, 40.02)	*U* = 636.00	0.003	0.006^*^
TMT-B	38.47 (29.53, 49.90)	26.73 (20.24, 35.29)	*U* = 646.50	0.002	0.005^*^
DSST	41.00 (33.25, 46.00)	55.00 (40.00, 67.00)	*U* = 254.00	0.007	0.008^*^
DST forward	8.00 (6.25, 9.00)	9.00 (8.00, 9.00)	*U* = 289.50	0.025	0.025^*^
DST backward	4.00 (4.00, 5.00)	5.00 (5.00, 6.50)	*U* = 249.00	0.004	0.006^*^
DST	12.00 (10.25, 14.00)	14.00 (13.00, 15.00)	*U* = 244.50	0.004	0.006^*^

Data presented as median (Q1, Q3) for non-normal distributions or mean ± SD for normal distributions. NPC, nasopharyngeal carcinoma; HCs, healthy controls; MoCA-B, Montreal cognitive assessment-basic; AVLT, auditory verbal learning test, AVLT (immediate) = immediate recall, AVLT (5 min) = short-term delayed recall, AVLT (20 min) = long-term delayed recall; TMT, trail making test; DSST, digit symbol substitution test; DST, digital span test.

^*^P < 0.05, which is considered statistically significant, with the false discovery rate (FDR) applied to correct for multiple comparisons.

### Gray matter volume alterations

**Figure 1:**
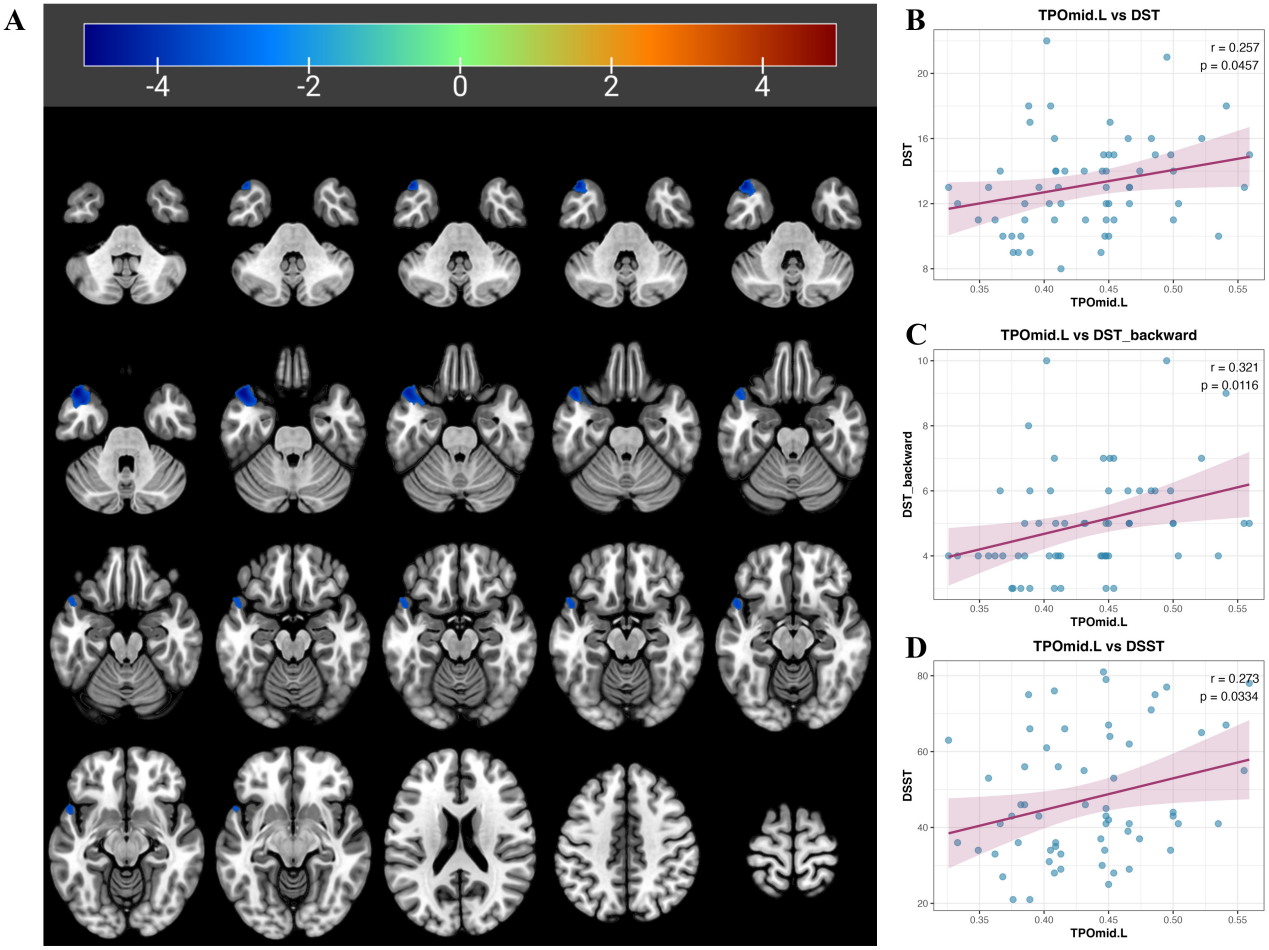
Gray matter (GM) volume alterations in VBM analysis and their correlation with cognitive scores. **(A)** Compared to healthy controls (HCs), the GM volume of the left temporal pole: middle temporal gyrus (TPOmid.L) is reduced in post-radiotherapy nasopharyngeal carcinoma (NPC) patients. Images of reduced GM volume (displayed in blue) are overlaid on an axial template. The color bar indicates *T*-values obtained from the two-sample *t*-test. **(B-D)** The GM volume of TPOmid.L are positive correlation with digit span test (DST), DST backword and digit symbol substitution test (DSST) of all participants without multiple comparison correction. However, these correlations do not remain statistically significant after multiple comparison correction. We can only posit that there exists a certain correlative trend between them.

In the VBM analysis, post-radiotherapy NPC patients exhibited significantly reduced GM volume only in the left temporal pole: middle temporal gyrus (TPOmid.L; *T* = −4.9982, cluster size = 447; Figure 1A). When no multiple comparison correction was applied, the GM volume of the TPOmid.L region showed positive correlations with scores on the DST, DST backward, and DSST (Figures 1B–D) of all participants. However, these correlations did not remain statistically significant after multiple comparison correction. We can only posit that there exists a certain correlative trend between them. No brain regions showed increased GM volume. In the subsample matched for age, sex, and education level, the GM volume of the TPOmid.L in NPC patients remained significantly reduced (*T* = −5.3108, cluster size = 318), with a voxel-level threshold of *P* < 0.001 and cluster size >100, presented in Supplementary Figure S1.

### Causal effects of gray matter volume patterns on TPOmid.L in NPC patients

**Figure 2:**
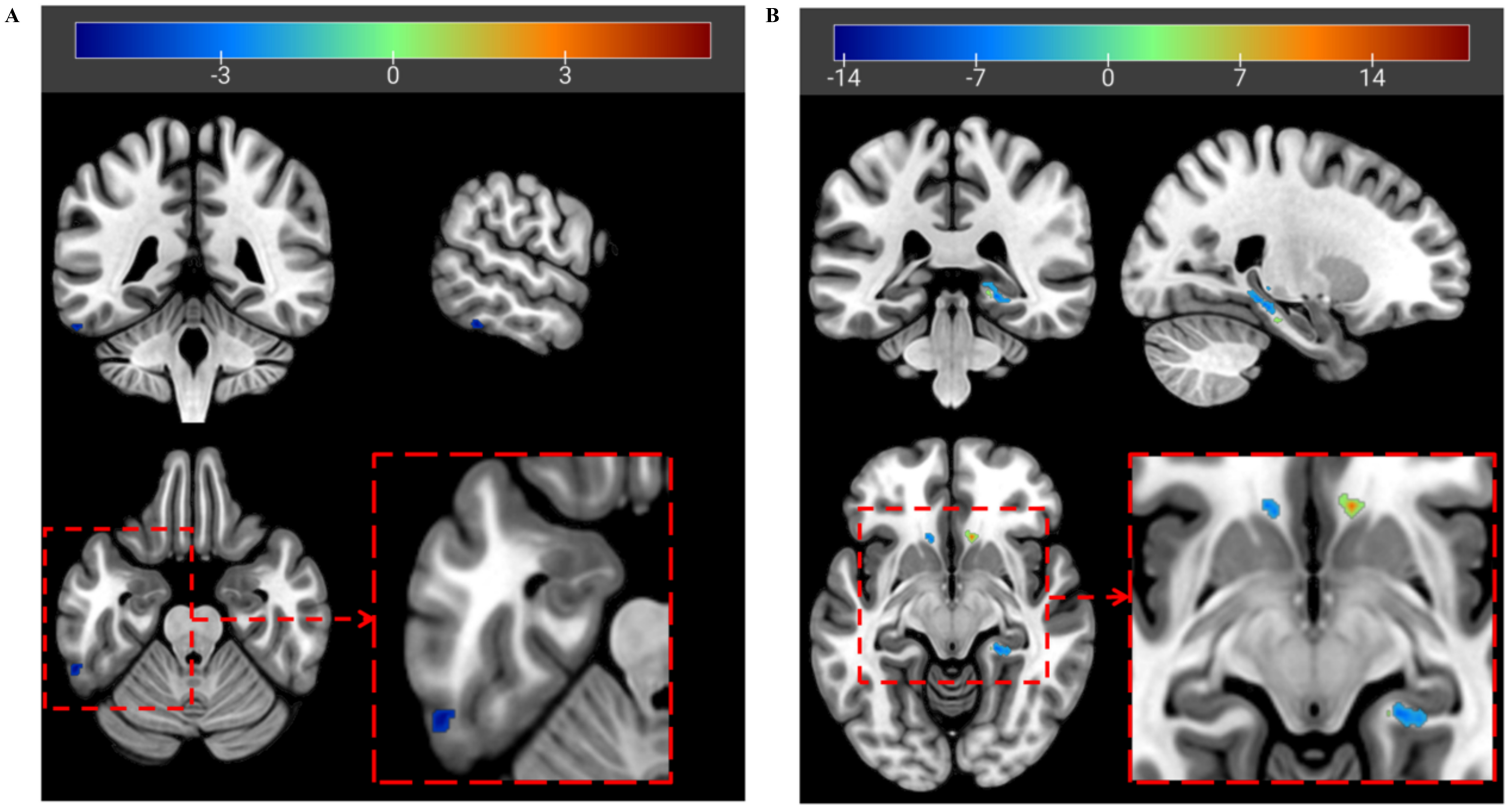
CaSCN analysis showing causal effects of gray matter (GM) pattern in post-radiotherapy NPC patients. **(A)** In the seed-to-map voxel wise Granger causality (GC) analysis, considering that the left temporal pole: middle temporal gyrus (TPOmid.L) demonstrated GM volume reduction in post-radiotherapy NPC patients, the left inferior temporal gyrus (ITG.L), with negative GC values, displayed an opposite GM volume alteration (i.e., volume increase) that lagged behind TPOmid.L atrophywhich may imply inverse-direction association. **(B)** In the map-to-seed voxel wise GC analysis, the TPOmid.L exhibited consistent GM volume changes directions with the right rectus gyrus (REC.R), left putamen (PUT.L), left precuneus (PCUN.L), and right cerebellar crus 1 (CC1.R), while showing opposite directions of GM volume changes compared to the the bilateral parahippocampal gyrus (PHG) and caudate nucleus (CAU). GC values were transformed to *Z* values. Color bar represents *Z* values from GC analysis, blue means negative *Z* values, red means positive *Z* values. The sign of the GC *Z* value indicates the direction of the modeled association under pseudo-time ordering: *Z* > 0 denotes a positive (same-direction) association, whereas *Z* < 0 denotes a negative (inverse-direction) association.

Given that TPOmid.L exhibited statistically significant between-group differences *via* VBM analysis, this region was chosen as the seed region. For mapping the causal effects associated with GM volume patterns in post-radiotherapy NPC patients, a CaSCN centered on TPOmid.L was constructed by performing GC analysis on morphometric data ordered according to the time after RT. In the seed-to-map voxel-wise GC analysis, no positive GC value was observed, although a negative GC value was identified exclusively in the left inferior temporal gyrus (ITG.L; Table 2 and Figure 2A). Considering that TPOmid.L demonstrated GM volume reduction in post-radiotherapy NPC patients, the ITG.L (with negative GC values) displayed an opposite GM volume alteration (i.e., volume increase) that lagged behind TPOmid.L atrophy, potentially indicating a inverse -direction association. In the map-to-seed voxel-wise GC analysis, positive GC values were concentrated in the right gyrus rectus (REC.R), left putamen (PUT.L), left precuneus (PCUN.L), and right cerebellar crus 1 (CC1.R), showing consistent GM volume change directions with the TPOmid.L (Table 2 and Figure 2B), which may imply same-direction association. Conversely, negative GC values were primarily observed in the bilateral parahippocampal gyrus (PHG) and caudate nucleus (CAU), exhibiting opposite GM change directions with the TPOmid.L (Table 2 and Figure 2B), which may imply inverse -direction association of the TPOmid.L on these regions. Besides to clarify the direction of GM volume changes of these regions, we additionally examined the association between regional GM volume and post-radiotherapy duration within the NPC patients after adjusting for age, sex, education, and Total intracranial volume (TIV). The results were presented in Supplementary Figure S2. These regions were subsequently used for ROI-to-ROI CaSCN analysis.

**Table 2:** Regions showing causal effect through voxel-wise CaSCN analysis in post-radiotherapy NPC patients.

Voxel-wise GC analysis	Region	Peak MNI coordinates			GC value	*Z* value	Cluster (Voxels)
		*X*	*Y*	*Z*			
Seed to map	ITG.L	−58.25	−43.75	−23.75	−1.86	−5.53	10
Map to seed	PHG.L	−24.25	−27.75	−17.75	−0.95	−9.40	12
	PHG.R	21.75	−35.75	−7.75	−0.99	−9.82	57
	CAU.L	−12.25	22.25	−9.75	−0.86	−8.56	11
	CAU.R	13.75	8.25	4.25	−1.47	−14.51	13
	REC.R	9.75	22.25	−9.75	1.40	13.63	17
	PUT.L	−14.25	6.25	4.25	1.96	19.13	19
	PCUN.L	−0.25	−45.75	58.25	0.71	6.83	16
	CC1.R	29.75	−63.75	−37.75	0.88	8.54	27

CaSCN, causal structural covariance network; GC, granger causality; MNI, Montreal neurologic institute; ITG, inferior temporal gyrus; PHG, parahippocampal gyrus; CAU, caudate nucleus; REC, rectus gyrus; PUT, putamen; PCUN, precuneus; CC1, cerebellar crus 1. The seed is left temporal pole: middle temporal gyrus (TPOmid.L), which exhibits gray matter (GM) volume atrophy in VBM analysis. The map is Anatomical Automatic Labeling (AAL) template, which includes cerebellar parcellations. The sign of the GC Z value indicates the direction of the modeled association under pseudo-time ordering: Z > 0 denotes a positive (same-direction) association, whereas Z < 0 denotes a negative (inverse-direction) association. Direction of GM volume change (↑/↓) is based on the adjusted association between regional GM volume and post-radiotherapy duration within the NPC patients (covariates: age, sex, education, and TIV). The results are presented in Supplementary Figure S2.

**Figure 3:**
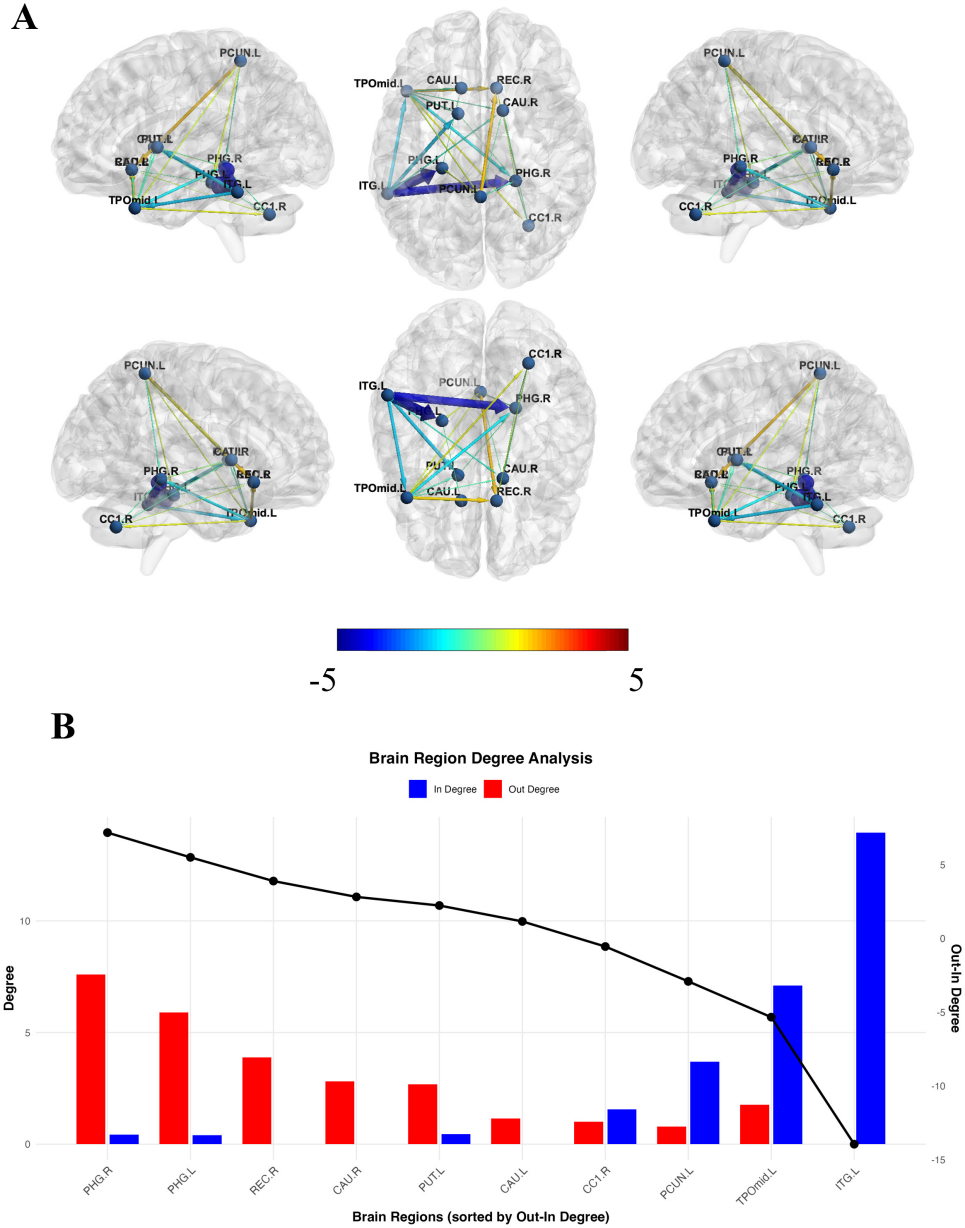
Regional causal structural covariance network (CaSCN) analysis shows causal relationships among ROIs. **(A)** Bivariate Granger causality (GC) analysis with signed path coefficients was employed to build an ROI-wise causal network characterizing inter-regional causal relationships. **(B)** The binary out- and in-degree values of each ROI were computed separately. Specifically, an ROI's binary in-degree value represents the sum of the number of paths projecting to the ROI and out-degree value reflects the sum of the number of paths projecting to other nodes. Furthermore, the binary out-in degree (out-degree minus in-degree) was calculated to identify the causal targets or causal source levels. Edge color denotes the sign of the GC *Z* value (positive vs. negative), representing same-direction vs. inverse statistical association under pseudo-time ordering. Edge thickness represents the magnitude of the *Z* value. Note that positive/negative *Z* values do not imply excitatory/inhibitory neurophysiological effects.

The ROI-wise CaSCN produced a directional network illustrating the causal relationships between the identified ROIs (Table 3 and Figure 3A). The bilateral PHG and CAU, along with REC.R and PUT.L, exhibited higher out-degree than in-degree values, indicating that these regions primarily project causal effects to others (Figure 3B). In contrast, CC1.R, PCUN.L, TPOmid.L, and ITG.L showed lower out-degree than in-degree values, suggesting these regions largely receive causal effects from other regions (Figure 3B). The TPOmid.L projected causal effects solely to the ITG.L and received causal effects from the PHG.R, REC.R, bilateral CAU, PUT.L, CC1.R and PCUN.L, which was identified to be transition point. In the multivariate GC analysis, the bilateral PHG and CAU, together with REC.R and PUT.L, demonstrated higher out-degree than in-degree values, whereas CC1.R, PCUN.L, TPOmid.L, and ITG.L showed the opposite pattern. These results broadly aligned with those from the bivariate GC analysis, as detailed in Supplementary Figure S3. Furthermore, within the subsample matched for age, sex, and education level, the bilateral PHG and CAU, along with REC.R and PUT.L, continued to exhibit higher out-degree than in-degree values, while CC1.R, PCUN.L, TPOmid.L, and ITG.L maintained lower out-degree than in-degree values, presented in Supplementary Figure S4.

**Table 3:** ROI-wise CaSCN analysis showing directional causal effect among the brain regions.

ROI-1	ROI-2	GC value	*Z* value
TPOmid.L	ITG.L	−1.76	−10.50
PHG.R	TPOmid.L	−1.54	−9.23
REC.R	TPOmid.L	1.63	9.44
CAU.L	TPOmid.L	−0.75	−4.58
CAU.R	TPOmid.L	−0.60	−3.68
PUT.L	TPOmid.L	−0.78	−4.73
CC1.R	TPOmid.L	1.01	5.81
PCUN.L	TPOmid.L	0.79	4.50
PHG.R	ITG.L	−4.39	−26.03
PHG.R	PCUN.L	−0.67	−4.06
PHG.R	CC1.R	−0.55	−3.35
PHG.R	PUT.L	−0.45	−2.78
PHG.L	ITG.L	−5.06	−29.97
PHG.L	PCUN.L	0.83	4.76
REC.R	PCUN.L	1.75	10.19
REC.R	CC1.R	0.51	2.87
CAU.L	PHG.L	−0.40	−2.49
CAU.R	ITG.L	−0.84	−5.06
CAU.R	CC1.R	−0.50	−3.10
CAU.R	PCUN.L	−0.45	−2.78
CAU.R	PHG.R	0.43	2.37
PUT.L	ITG.L	−1.90	−11.33

ROI, region-of-interest; CaSCN, causal structural covariance network; left temporal pole: middle temporal gyrus, TPOmid.L; ITG, inferior temporal gyrus; PHG, parahippocampal gyrus; REC, rectus gyrus; CAU, caudate nucleus; PUT, putamen; PCUN, precuneus; CC1, cerebellar crus I. Higher GC values indicate stronger causal effects.

## Discussion

Through a study of T1-weighted structural data from patients with radiation-induced brain injury (RBI) using causal structural covariance network (CaSCN) combined with Granger causality (GC) analysis, we evaluated the regulatory relationships between brain regions with progressive gray matter (GM) structural damage. For the first time, we revealed the regulatory pattern of brain structural injury from a “source-sink” perspective: the bilateral parahippocampal gyrus (PHG), the right gyrus rectus of the frontal lobe (REC.R) and the right caudate nucleus (CAU.R) exhibit typical source-like characteristics, as their structural changes actively regulate and influence GM volume alterations in other brain regions; in contrast, the left precuneus (PCUN.L), the left temporal pole, middle temporal gyrus (TPOmid.L), and inferior temporal gyrus (ITG.L) show distinct sink-like features, mainly receiving regulation and influence from source-like brain regions and serving as key target regions of structural damage. This finding provides a novel regulatory framework for understanding the progressive pathological process of RBI. Previous RBI studies have demonstrated that NPC patients who received RT exhibit reduced GM volumes in multiple brain regions (Lv et al., 2014), including the temporal lobe, frontal lobe, parietal lobe, and cerebellum, which are generally consistent with the results of the present study.

The results of this study indicate that the temporal changes in brain structure can be evaluated by applying Granger causality (GC) analysis to morphometric data categorized according to the time after RT. By integrating information on disease duration, pseudo-time series of disease progression and lifespan-related information can be generated. The CaSCN analysis approach reveals that structural changes in one region influence those in another. These results further suggest that CaSCN analysis can serve as an effective method to investigate the interregional covariance of structural changes in patients with RBI.

In the present study, we found that the global GM volume shows a progressive trend of change with the extension of post-radiotherapy time. These results further confirm the “source-sink”—based regulatory relationships among brain regions. The study clearly demonstrates that the PHG.L, PHG.R, REC.R, and CAU.R as core source-like brain regions, serve as key hubs of the directed network and exert active regulatory effects on multiple regions including the basal ganglia, cerebellum, parietal lobe, and temporal lobe. In contrast, the PCUN.L, TPOmid, and ITG served as the primary sink-like brain regions, receiving regulatory effects from source-like regions such as the PHG. Collectively, our findings indicate that source-like regions exhibited potential regulatory associations with GM damage in target regions such as the temporal lobes. This source-sink regulatory pattern clearly illustrates the progressive pathological process of structural injury in RBI.

Previous studies have demonstrated that the hippocampus exhibited GM atrophy in the early stage after RT, and this atrophy progresses progressively with the passage of time post-radiotherapy (Lv et al., 2019), showing a time-dependent pattern. In our study, the PHG is the most critical source-like brain region in RBI. Its GM volume change exerts positive regulatory effects on multiple regions, including the basal ganglia, cerebellum, parietal lobe, and temporal lobe, and serves as the core hub of the directed network, which is consistent with previous studies. Pathologically, the hippocampus is particularly sensitive to acute and subacute radiation damage (Ren et al., 2021), and cognitive impairments such as deficits in attention, memory, and executive function during this period are associated with hippocampal damage (Son et al., 2015); studies have also found that early radiation-induced damage is closely related to vascular injury, manifested as vascular dilation, vascular endothelial cell damage, and increased vascular wall permeability, and significant vascular damage can be observed in the hippocampus following radiation exposure (Makale et al., 2017). This also explains why structural changes in the parahippocampal gyrus can dominate and influence the injury progression of other subsequent brain regions. Animal experiments have revealed a significant increase in the gray matter volume of the right hippocampal dentate gyrus region in mice after irradiation (Zhu et al., 2025). The PHG is located on the medial side of the hippocampus, while the hippocampus itself is situated in the medial temporal lobe—an area typically within the radiation field that exposes NPC patients to unnecessary radiation (Mao et al., 2014)—and when designing radiotherapy plans for NPC patients, the hippocampus is generally not considered one of the organs at risk (OARs; Sun et al., 2014). The source-like regulatory role of the parahippocampal gyrus not only help explain the pathogenesis of cognitive impairments (e.g., memory and attention decline) after radiotherapy but also reveal the critical role of hippocampal damage in post-radiotherapy brain structural injury; therefore, efforts should be made to minimize radiation damage to the hippocampus to delay the occurrence of subsequent injuries, providing a basis for designing appropriate hippocampal dose reduction strategies.

In addition to other regions, the gyrus rectus (REC) of the frontal lobe also projects regulatory effects to brain areas such as the cerebellum, parietal lobe, and temporal lobe. Studies by Lv et al. (2014) also observed a significant reduction in GM volume of the frontal lobe after RT (Lv et al., 2014). Given that the frontal lobe is relatively far from the radiation field, the researchers proposed that the GM damage in the frontal lobe post-radiotherapy might be attributed to chemotherapy. It is generally accepted that structural damage often leads to abnormalities in functional networks. Our previous brain function studies (Leng et al., 2021), along with other prior research (Fu et al., 2022) have found that the functional connectivity of the frontal lobe is significantly impaired after radiotherapy—with multiple functional connections showing a marked decrease—and this impairment is associated with various cognitive disorders. The frontal lobe is a component of both the default mode network (DMN) and the central executive network (CEN), and it is involved in higher-order cognitive processes (Astle et al., 2015). Studies by Lin et al. have indicated that the GM structures of brain regions including the frontal lobe within the DMN was susceptible to radiation damage (Lin et al., 2017); however, this damage is dynamic and transient. Impairment of frontal lobe function may result in cognitive deficits, particularly in working memory and executive function.

This study also found that the basal ganglia, including the caudate nucleus andputamen, can project regulatory effects to brain regions such as the temporal lobe. Previous studies have revealed a significant reduction in thalamic GM volume after RT (Deng et al., 2024) and have also observed pathological changes such as gliosis and vascular thickening in the putamen following cranial radiotherapy (Wick et al., 2000). The pathogenesis of RBI includes direct damage to brain tissue by radiation, vascular injury, and immune inflammatory responses. Since the basal ganglia are rich in neurons and blood vessels, these pathological processes are likely to affect the basal ganglia. For example, radiation-induced increases in cerebrovascular permeability and thrombus formation can impair blood supply to the basal ganglia, leading to local ischemia and hypoxia, which in turn trigger neuronal damage and other changes. Another study indicated that the decoupling between GM structure and function in brain regions such as the putamen was closely associated with RBI and may lead to the decline of motor function (Kang et al., 2022).

The cerebellum is also one of the brain regions susceptible to RBI identified in previous studies (Shen et al., 2016). Research has shown that significant abnormalities occur in both the function and GM structure of the cerebellum after radiotherapy, and these abnormalities are associated with sensory and motor dysfunctions (Kang et al., 2022). Additionally, Ma et al. observed a marked reduction in cerebellar functional connectivity post-radiotherapy, and this reduction was closely correlated with cognitive impairments (Ma et al., 2016). Deng et al. found that the gray matter volume in multiple cerebellar regions was significantly reduced in patients after radiotherapy, and the researchers suggested that the regional reduction in cerebellar gray matter volume may lead to the disruption of structural covariation among different cerebellar subregions (Deng et al., 2024). Our previous research also revealed a significant decrease in cerebellar GM volume in the early stage after radiotherapy, which was associated with memory and executive function deficits (Qin et al., 2022). Previous studies have often suggested that the cerebellum is relatively close to the radiation field, making it vulnerable to direct radiation damage (Du, 2024); however, the results of the present study indicat that cerebellar damage may partially occur secondarily to injuries in brain regions such as the PHG and frontal lobe.

In the present study, the precuneus also received projections of significant regulatory effects. Previous studies have shown that changes in the function and GM structure of the precuneus exhibit excellent classification performance in predicting RBI (Kang et al., 2022). The precuneus is part of the superior parietal lobule, and there have been numerous reports of similar findings regarding post-radiotherapy GM damage in the parietal lobe. Lv et al. conducted a study on brain GM volume in NPC patients after radiotherapy using 3D-T1WI and found a significant reduction in GM volume of the right inferior parietal lobule in these patients; at that time, the researchers suggested that, similar to the frontal lobe, parietal lobe damage after radiotherapy was difficult to explain and might be partially attributed to the effects of chemotherapy (Lv et al., 2014). In contrast, Lin et al. observed that compared with the pre-radiotherapy group, the late-delayed reaction group after radiotherapy showed a significant increase in GM thickness of the bilateral inferior parietal lobule; the researchers hypothesized that compensatory changes might occur in the parietal lobe during the late-delayed reaction phase, and these changes were associated with various cognitive impairments (Lin et al., 2017). The results of the present study indicate that parietal lobe damage may be secondary damage occurring as a consequence of injuries in brain regions such as the PHG, frontal lobe, and basal ganglia.

Previous studies have found that changes in the function-GM structure coupling of the temporal lobe after radiotherapy are relatively minor (Kang et al., 2022), and this phenomenon was thought to be inhibited by the function-GM structure coupling of brain regions such as the precuneus—consistent with the results of the present study. At that time, researchers considered the pathogenesis of this phenomenon unclear, while the present study suggests that it may be due to the precuneus projecting regulatory effects to the temporal lobe. All brain structure studies on RBI have shown that the temporal lobe is a target brain region for RBI (Lin et al., 2021; Pan et al., 2024). Our previous research also revealed a significant reduction in GM volume of the temporal pole, MTG, and ITG after RT, which was associated with various cognitive impairments such as memory deficits (Qin et al., 2022). The present study demonstrates that the TPOmid.L, and ITG.L receive projections of regulatory effects from brain regions including the PHG, frontal lobe, and basal ganglia. This implies that in addition to direct radiation-induced damage, the temporal lobe may also experience secondary damage secondary to injuries in other radiation-sensitive brain regions. This finding suggests that we should protect radiation-sensitive brain regions such as the PHG to reduce secondary damage to the temporal lobe, thereby delaying or reducing the occurrence of temporal lobe RBI.

Our study has several limitations. First, the underlying physiological mechanisms of disease progression remain unclear. Additionally, time-organized data cannot directly reflect the true temporal sequence of disease progression; therefore, longitudinal studies must be conducted to further clarify the causal relationships between structural defects. Second, although age was regressed as a covariate in the CaSCN analysis, its confounding effect on structural images cannot be completely excluded, and the causal relationship between structural changes and disease course should be interpreted with caution. Third, although all patients received chemotherapy, the heterogeneity of specific regimens and the limited subgroup sizes prevented stratified analyses. Therefore, the potential contribution of chemotherapy to brain structural alterations cannot be fully excluded. Furthermore, the approach of dividing all patients into several subgroups based on disease course stages for stage-specific comparisons is an arbitrary strategy. Finally, the validity of the CaSCN analysis results needs to be verified using a replication sample.

## Conclusion

The causal structural covariance network (CaSCN) analysis approach was used to evaluate the interregional regulatory effects of structural changes and disease progression in patients with NPC after RT. The PHG.L, PHG.R, REC.R and CAU.R are core source-like brain regions, and their structural changes exert a key regulatory effect on GM volume alterations across multiple brain regions. The PCUN.L, TPOmid.L, and ITG.L show distinct sink-like features, which mainly receive regulatory effects from source-like brain regions, acting as major target regions of structural damage. These findings imply a hierarchical structure of structural brain injury, as well as the important roles of the parahippocampal gyrus, gyrus rectus and caudate nucleus in the progression of RBI.Our work provides further evidence that RBI is associated with progressive GM abnormalities.

## Data Availability

The raw data supporting the conclusions of this article will be made available by the authors, without undue reservation.
