# Supplementary material for: Causal network analysis-based assessment of gray matter alteration in post-radiotherapy nasopharyngeal carcinoma patients using 3D T1-weighted MRI
Source: Front Neurosci. 2026 Apr 13;20:1709659. doi: 10.3389/fnins.2026.1709659 (PMC13111192; doi:10.3389/fnins.2026.1709659)
Supplement: Supplementary file 1 [file Supplementary_file_1.docx]

**Supplementary Material**


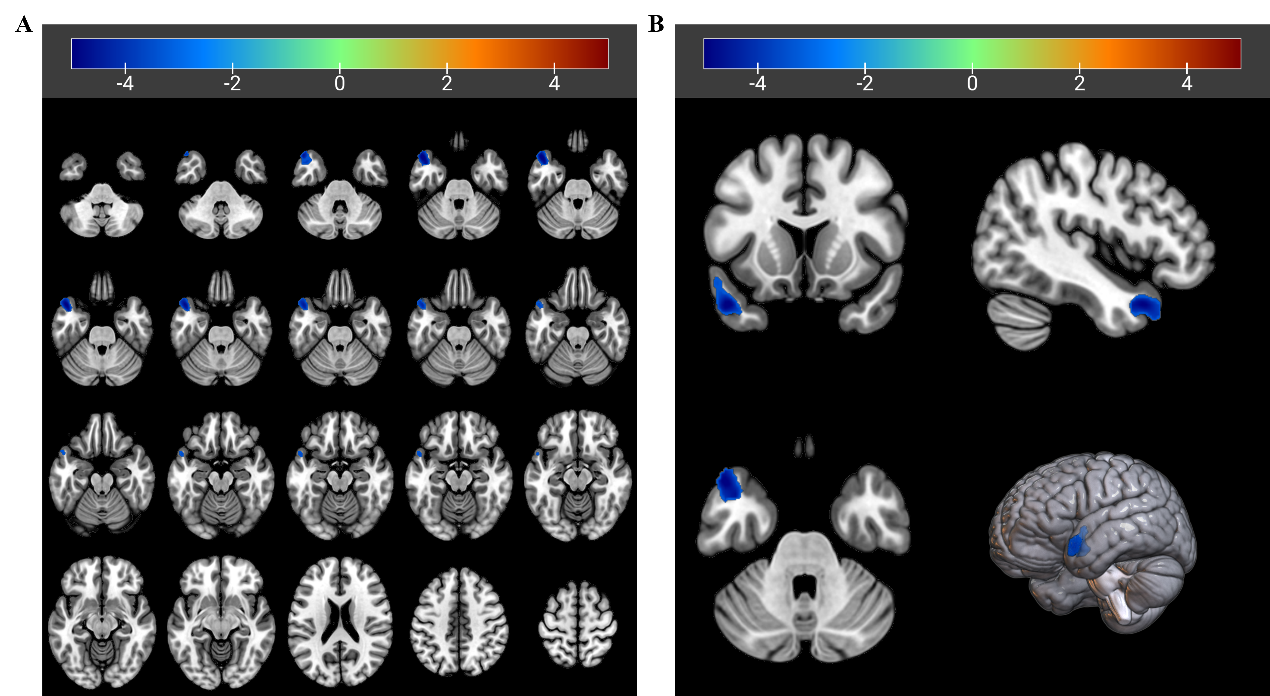


**Figure S1**: Gray matter (GM) volume alterations in VBM analysis of the subsample (21 NPC patients vs 23 HCs). Compared to healthy controls (HCs), the GM volume of the left temporal pole: middle temporal gyrus (TPOmid.L) is reduced in post-radiotherapy NPC patients. Images of reduced GM volume (displayed in blue) are overlaid on an axial template (A) and in a three-dimensional (3D) template (B). The color bar indicates T-values obtained from the two-sample t-test.


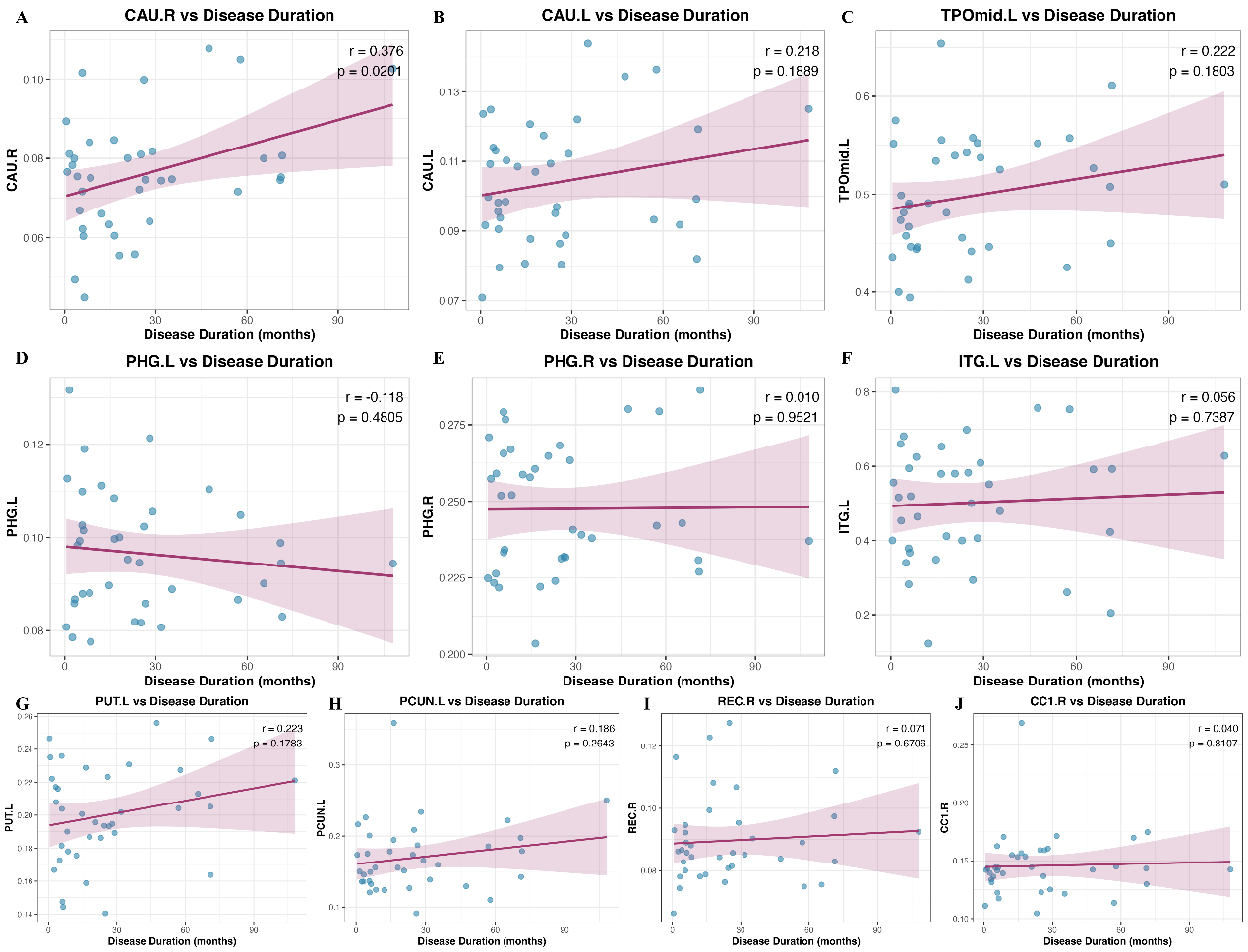


**Figure S2:** The association between regional GM volume and post-radiotherapy duration within the NPC patients after adjusting for age, sex, education, and Total intracranial volume (TIV). (A) The GM volume of the CAU.R shows a significantly positive correlation with post-radiotherapy duration. (B-J) The GM volume of the CAU.L, TPOmid.L, PHG.L, PHG.R, ITG.L, PUT.L, PCUN.L, REC.R and CC1.R show no significantly correlation with post-radiotherapy duration. However, the GM volume of the PHG.L exhibit a trend toward a negative correlation with post‑radiotherapy duration, while positive correlative trends were observed for the other regions.


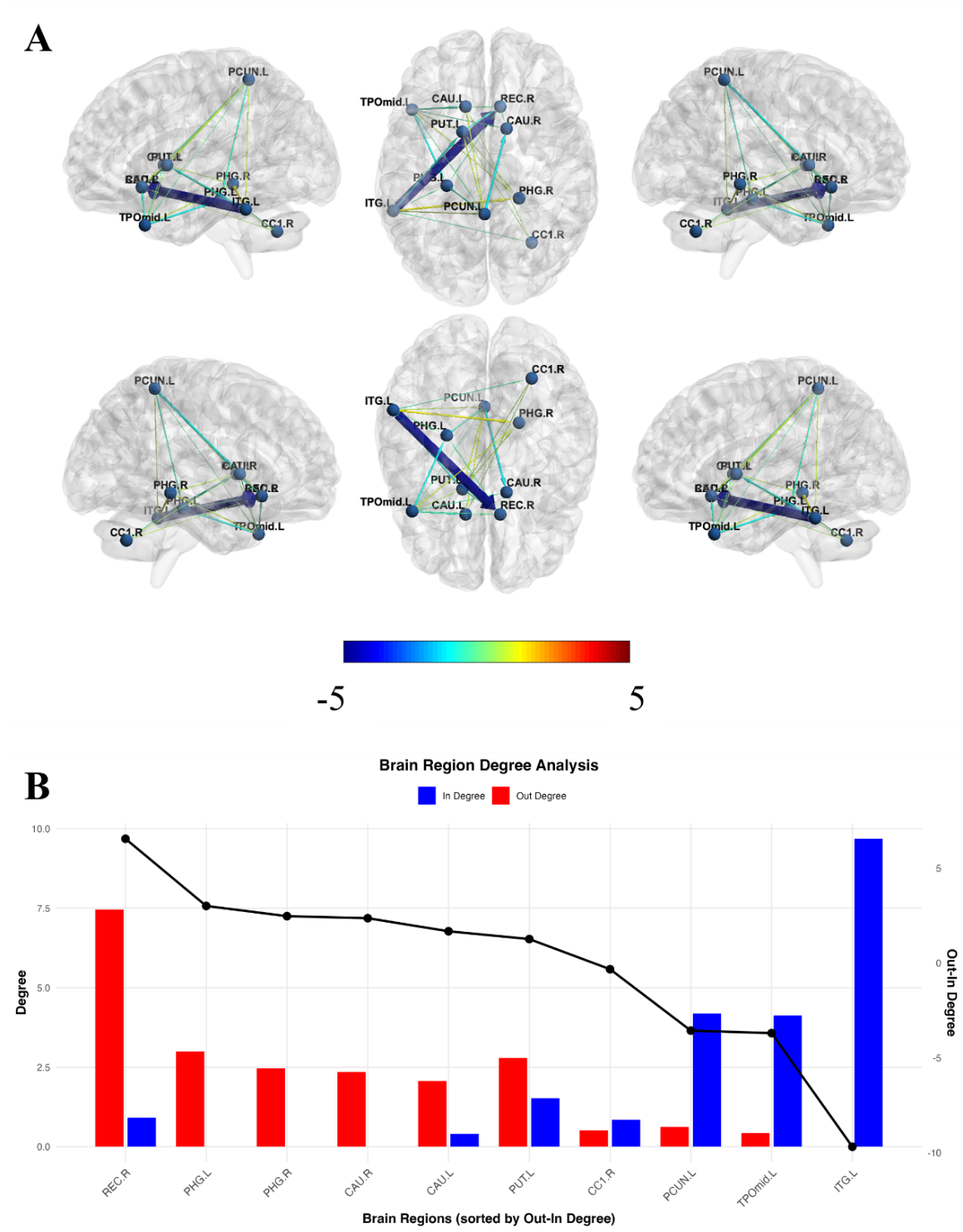


**Figure S3:** Regional causal structural covariance network (CaSCN) analysis shows causal relationships among ROIs in the multivariate Granger causality analysis. (A) Bivariate Granger causality (GC) analysis with signed path coefficients was employed to build an ROI-wise causal network characterizing inter-regional causal relationships. (B) The binary out- and in-degree values of each ROI were computed separately. Specifically, an ROI's binary in-degree value represents the sum of the number of paths projecting to the ROI and out-degree value reflects the sum of the number of paths projecting to other nodes. Furthermore, the binary out-in degree (out-degree minus in-degree) was calculated to identify the causal targets or causal source levels. In the multivariate GC analysis, the bilateral PHG and CAU, together with REC.R and PUT.L, demonstrated higher out-degree than in-degree values, whereas CC1.R, PCUN.L, TPOmid.L, and ITG.L showed the opposite pattern. These results broadly aligned with those from the bivariate GC analysis. Edge color denotes the sign of the GC Z value (positive vs negative), representing same-direction vs inverse statistical association under pseudo-time ordering. Edge thickness represents the magnitude of the Z value. Note that positive/negative Z values do not imply excitatory/inhibitory neurophysiological effects.


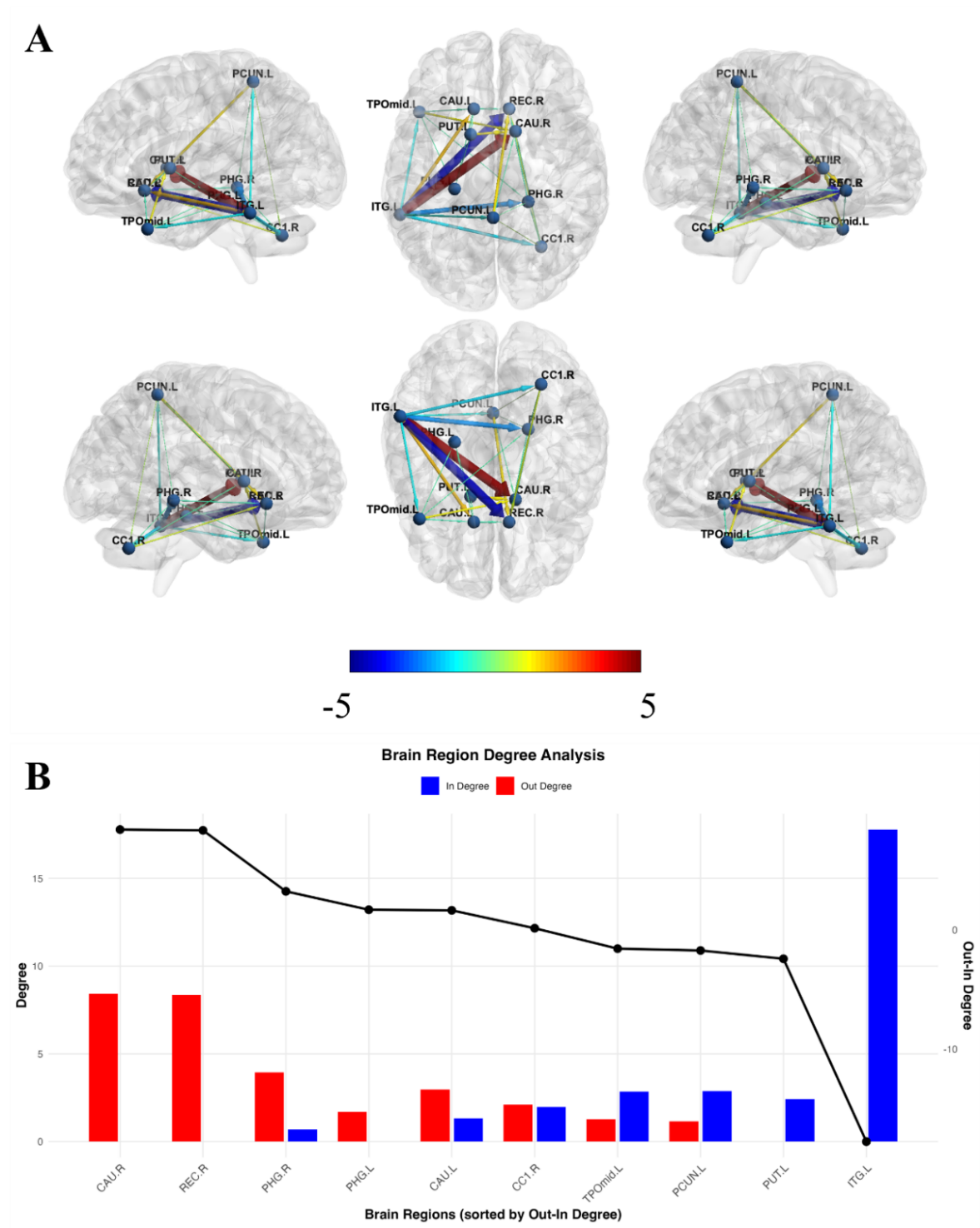


**Figure S4:** Regional causal structural covariance network (CaSCN) analysis shows causal relationships among ROIs in the subsample. (A) Bivariate Granger causality (GC) analysis with signed path coefficients was employed to build an ROI-wise causal network characterizing inter-regional causal relationships. (B) The binary out- and in-degree values of each ROI were computed separately. Specifically, an ROI's binary in-degree value represents the sum of the number of paths projecting to the ROI and out-degree value reflects the sum of the number of paths projecting to other nodes. Furthermore, the binary out-in degree (out-degree minus in-degree) was calculated to identify the causal targets or causal source levels. In the subsample matched for age, sex, and education level, the bilateral PHG and CAU, along with REC.R and PUT.L, continued to exhibit higher out-degree than in-degree values, while CC1.R, PCUN.L, TPOmid.L, and ITG.L maintained lower out-degree than in-degree values. Edge color denotes the sign of the GC Z value (positive vs negative), representing same-direction vs inverse statistical association under pseudo-time ordering. Edge thickness represents the magnitude of the Z value. Note that positive/negative Z values do not imply excitatory/inhibitory neurophysiological effects.
