# Supplementary material for: Causal network analysis-based assessment of gray matter alteration in post-radiotherapy nasopharyngeal carcinoma patients using 3D T1-weighted MRI
Source: Front Neurosci. 2026 Apr 13;20:1709659. doi: 10.3389/fnins.2026.1709659 (PMC13111192; doi:10.3389/fnins.2026.1709659)
Supplement: Supplementary file 2 [file Table_1.docx]

**Supplementary Material**

| TABLE S1\| Clinical characteristics and cognitive assessment of post-radiotherapy NPC patients and HCs in the subsample | | | | | |
| --- | --- | --- | --- | --- | --- |
| Clinical information | **NPC patients(n=21)** | **HCs(n=23)** | **Statistics** | **P-value** | **FDR-corrected** |
| Clinical characteristics |  |  |  |  |  |
| Age(years) | 42.00(38.00,50.00) | 37.00(28.50,52.00) | U=282.00 | 0.347 | 0.586 |
| Sex(M/F) | 16/5 | 16/7 | χ²= 0.02 | 0.878 | 0.878 |
| Education level(years) | 12.00(12.00,15.00) | 15.00(12.00,16.00) | U=206.00 | 0.391 | 0.586 |
| Cognitive scores |  |  |  |  |  |
| MoCA-B | 25.00(25.00,27.00) | 28.00(27.00,29.00) | U=88.50 | ＜0.001 | 0.003* |
| AVLT (immediate) | 21.43±3.38 | 25.35±4.86 | t=-3.08 | 0.004 | 0.013* |
| AVLT (5min) | 9.00(7.00,10.00) | 10.00(8.50,11.00) | U=154.00 | 0.038 | 0.052 |
| AVLT (20min) | 8.00(7.00,9.00) | 10.00(8.50,11.00) | U=115.50 | 0.003 | 0.013^*^ |
| AVLT (recognition) | 11.00(10.00,12.00) | 12.00(11.50,12.00) | U=186.00 | 0.139 | 0.139 |
| TMT-A | 41.55(27.84,58.23) | 29.96(25.20,40.02) | U=327.00 | 0.046 | 0.056 |
| TMT-B | 38.40(29.45,50.42) | 26.73(20.24,35.29) | U=349.50 | 0.012 | 0.025^*^ |
| DSST | 39.00(30.00,43.00) | 55.00(40.00,67.00) | U=129.00 | 0.008 | 0.023^*^ |
| DSTforward | 8.00(7.00,9.00) | 9.00(8.00,9.00) | U=167.50 | 0.075 | 0.082 |
| DST backward | 4.00(4.00,5.00) | 5.00(5.00,6.50) | U=152.50 | 0.033 | 0.051 |
| DST | 12.71±2.28 | 14.35±2.59 | t=-2.21 | 0.032 | 0.051 |
| Data presented as median(Q1,Q3) for non-normal distributions or mean±SD for normal distributions. NPC, nasopharyngeal carcinoma; HCs, healthy controls; MoCA-B, Montreal cognitive assessment-basic; AVLT, auditory verbal learning test, AVLT (immediate) = immediate recall, AVLT (5min) = short-term delayed recall, AVLT (20min) = long-term delayed recall; TMT, trail making test; DSST, digit symbol substitution test; DST, digital span test.  *P < 0.05, which is considered statistically significant, with the false discovery rate (FDR) applied to correct for multiple comparisons. | | | | | |
