# Supplementary figures and images for: Causal network analysis-based assessment of gray matter alteration in post-radiotherapy nasopharyngeal carcinoma patients using 3D T1-weighted MRI
Source: Front Neurosci. 2026 Apr 13;20:1709659. doi: 10.3389/fnins.2026.1709659 (PMC13111192; doi:10.3389/fnins.2026.1709659)

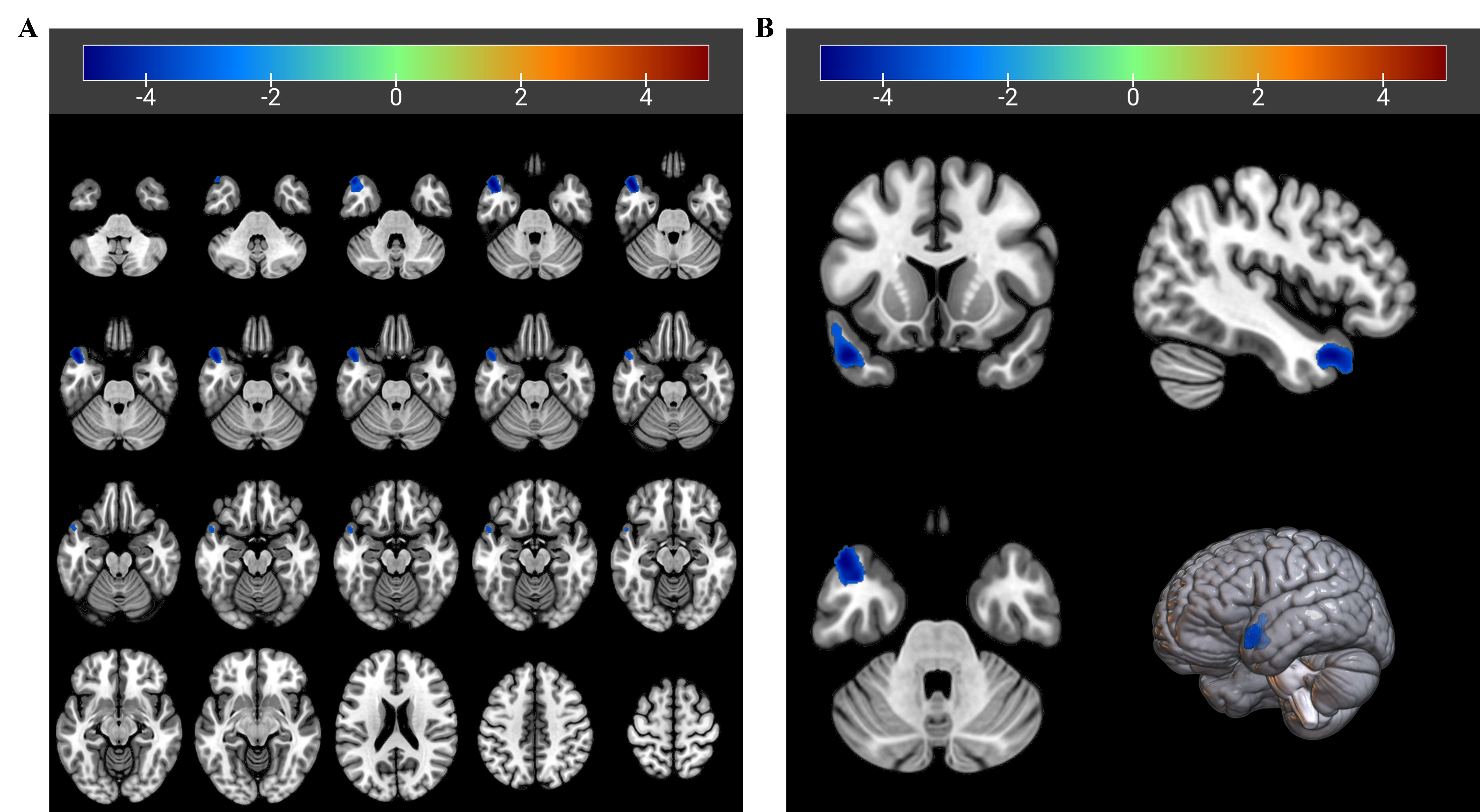

Supplement: Supplementary file 3 [file Image_1.tif]

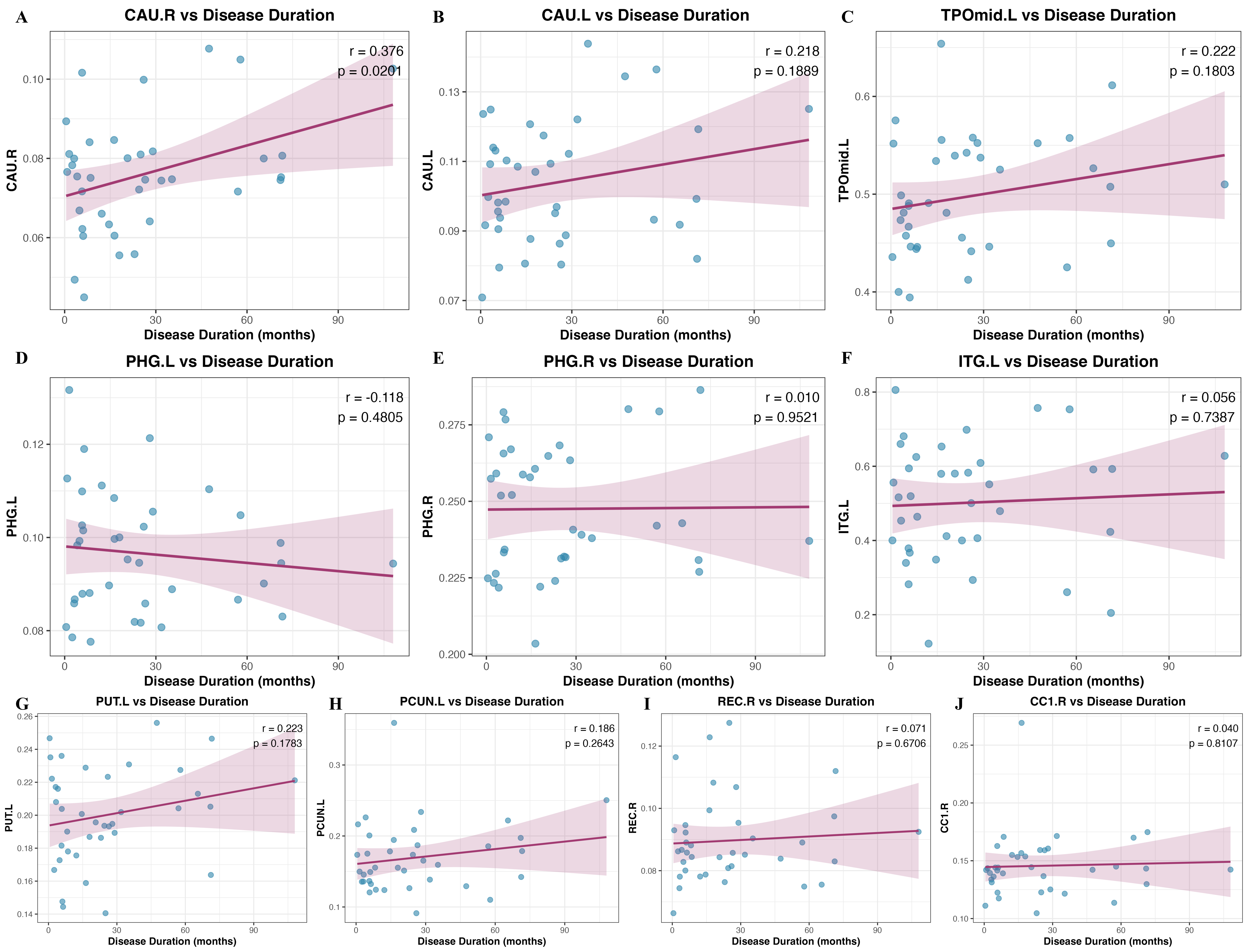

Supplement: Supplementary file 4 [file Image_2.tif]

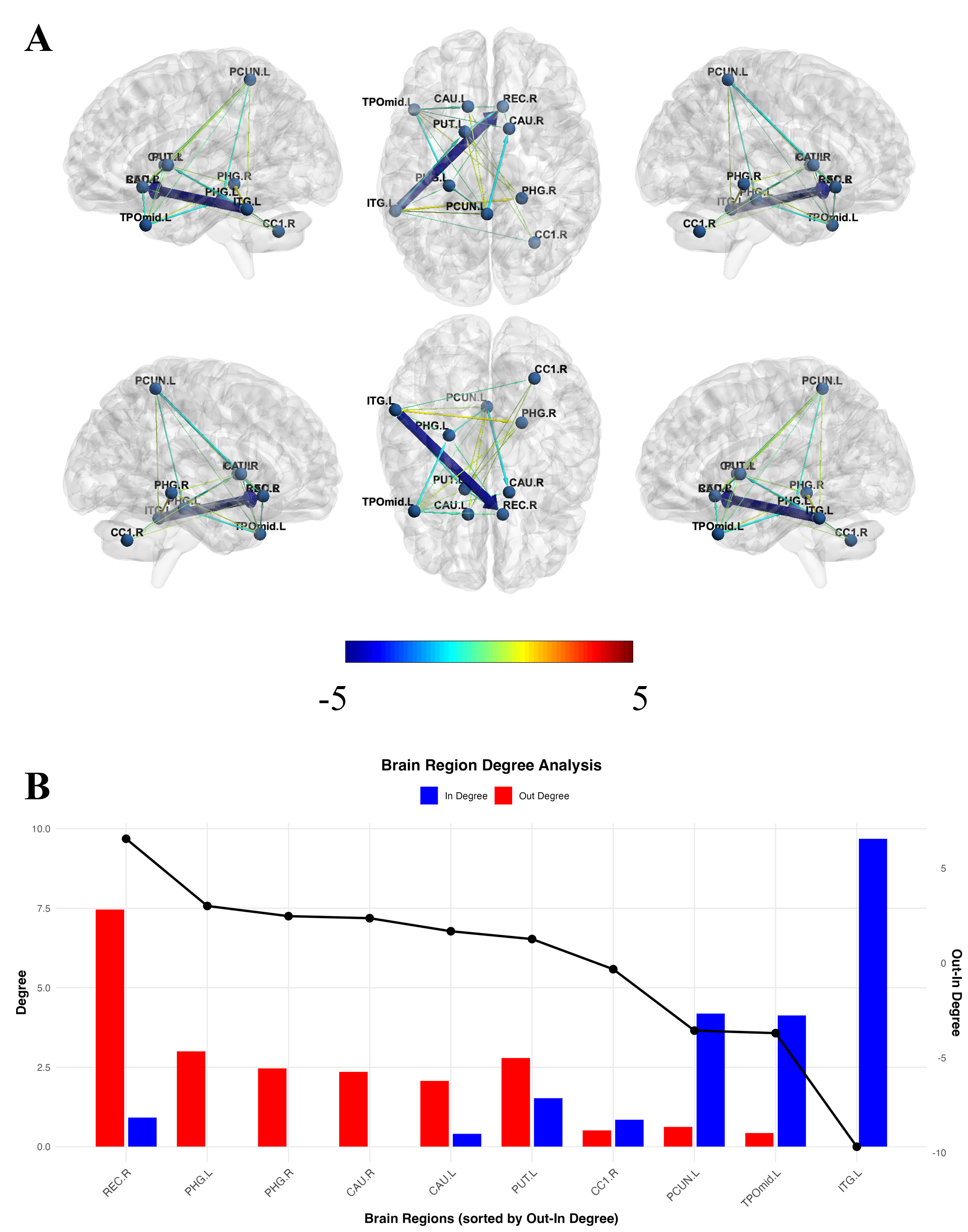

Supplement: Supplementary file 5 [file Image_3.tif]

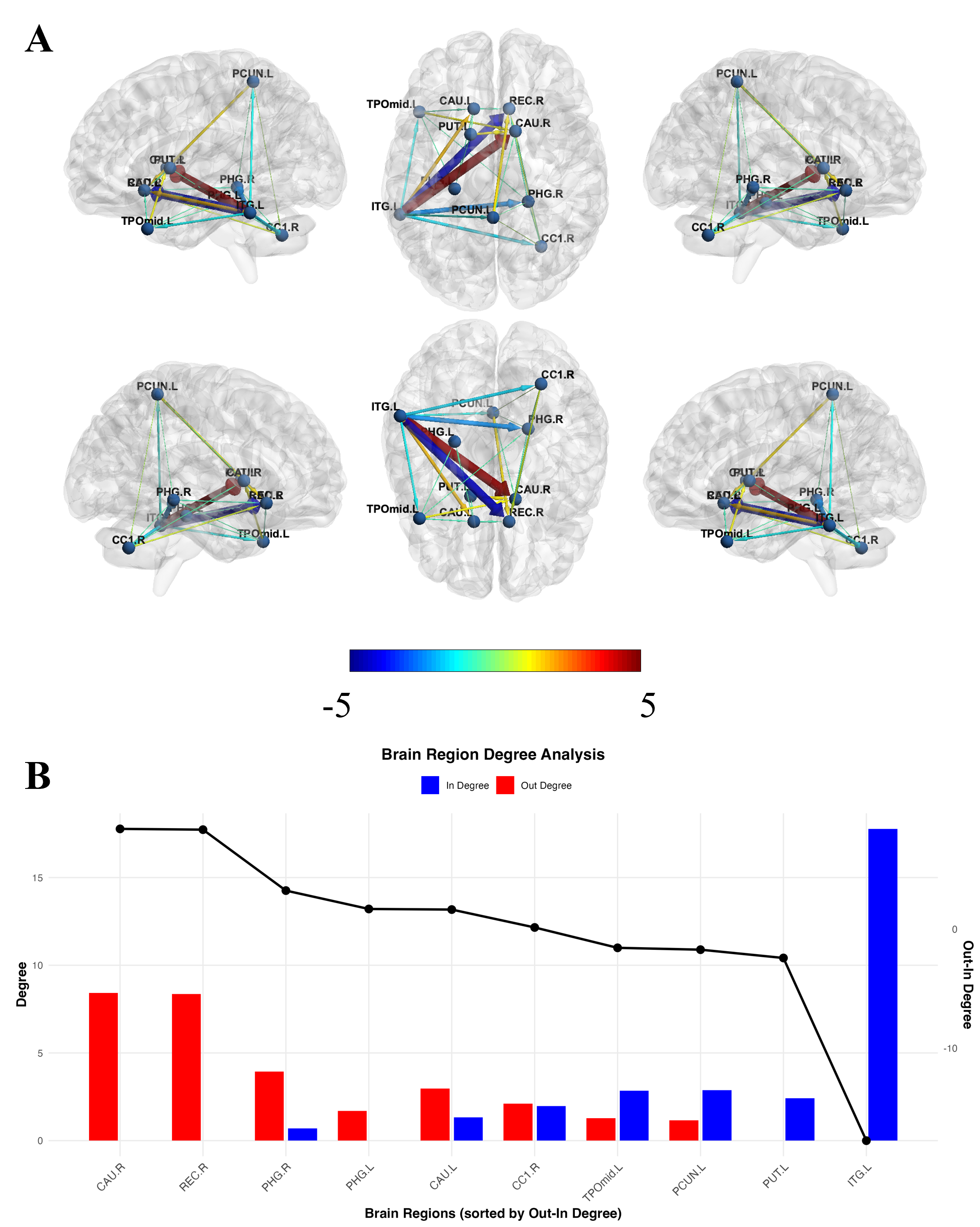

Supplement: Supplementary file 6 [file Image_4.tif]
